# Multiplexed Quantitative Assessment of the Fate of Taurine and Sulfoquinovose in the Intestinal Microbiome

**DOI:** 10.3390/metabo10110430

**Published:** 2020-10-26

**Authors:** Sven-Bastiaan Haange, Nicole Groeger, Jean Froment, Theresa Rausch, Wiebke Burkhardt, Svenja Gonnermann, Annett Braune, Michael Blaut, Martin von Bergen, Ulrike Rolle-Kampczyk

**Affiliations:** 1 Department of Molecular Systems Biology, Helmholtz Centre for Environmental Research—UFZ, 04318 Leipzig, Germany; nicole.groeger@ufz.de (N.G.); Jean.Froment@aces.su.se (J.F.); martin.vonbergen@ufz.de (M.v.B.); 2 Research Group Intestinal Microbiology, Department of Molecular Toxicology, German Institute of Human Nutrition Potsdam-Rehbruecke, 14558 Nuthetal, Germany; Theresa.Rausch@dife.de (T.R.); Wiebke.Burkhardt@dife.de (W.B.); sgonnermann@t-online.de (S.G.); braune@dife.de (A.B.); blaut@dife.de (M.B.); 3 Institute of Biochemistry, Faculty of Life Sciences, University of Leipzig, 04103 Leipzig, Germany

**Keywords:** liquid chromatography, mass spectrometry, multi-reaction monitoring, quantification, sulfonates, taurine, sulfoquinovose, intestinal microbiome

## Abstract

(1) Introduction: Sulfonates, which can be diet- or host-derived, are a class of compounds detected in the gut, are involved in host–microbiome interactions and have several health effects. Our aim was to develop a method to quantify five of the sulfonates in the intestine and apply it in a simplified human microbiome model. These were taurine, its metabolic precursor cysteate and one of its degradation products isethionate, as well as sulfoquinovose and one of its most relevant degradation products 2,3-dihydroxy-1-propanesulfonate. (2) Methods: An extraction and sample preparation method was developed, without the need for derivatization. To detect and quantify the extracted sulfonates, a multiplexed LC-MS/MS-MRM method was established. (3) Results: The accuracy and precision of the method were within GLP-accepted parameters. To apply this method in a pilot study, we spiked either taurine or sulfoquinovose into an in vitro simplified human microbiota model with and without *Bilophila wadsworthia*, a known sulfonate utilizer. The results revealed that only the culture with *B. wadsworthia* was able to degrade taurine, with isethionate as an intermediate. After spiking the communities with sulfoquinovose, the results revealed that the simplified human microbiome model was able to degrade sulfoquinovose to 2,3-dihydroxypropane-1-sulfonate, which was probably catalyzed by *Escherichia coli*. In the community with *B. wadsworthia*, the 2,3-dihydroxypropane-1-sulfonate produced was further degraded by *B. wadsworthia* to sulfide. (4) Conclusions: We successfully developed a method for sulfonate quantification and applied it in a first pilot study.

## 1. Introduction

In recent years, the importance of the interactions between the intestinal microbiome and host in human health and disease has emerged [1,2]. These interactions are mainly conveyed by the exchange of chemical compounds and their metabolites. An important class of compounds detected in the gut are the sulfonates. Sulfonates and their final degradation product, H_2_S, have been linked to many host functions as well as beneficial and detrimental health effects [3,4].

**Figure 1:**
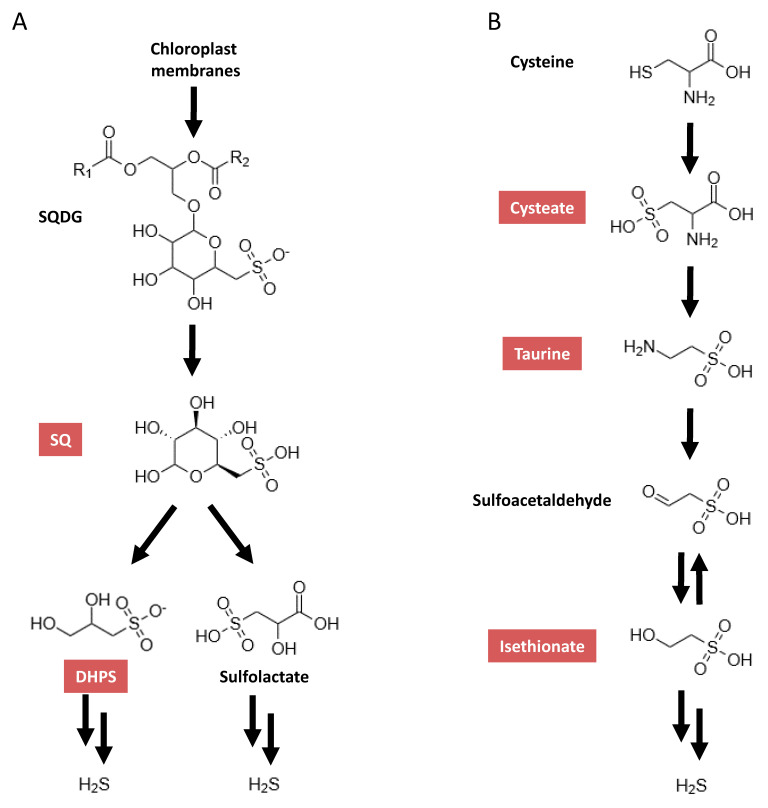
Simplified metabolic pathways for taurine (modified from Kyoto Encyclopedia of Genes and Genomes (KEGG) pathway map 00430) (**A**) and sulfoquinovose (modified from Denger et al. [6] and Felux et al. [16]) (**B**). SQDG, sulfoquinovosyl diacylglycerols; SQ, sulfoquinovose; DHPS, 2,3-dihydroxy-1-propanesulfonate. Compounds quantifiable by the presented method are highlighted in red.

One group of sulfonates are the sulfoquinovosyl diacylglycerols (SQDG), which are a class of sulfolipids and are major constituents of chloroplast membranes [5]. Thus, a diet rich in green leafy vegetables will include a high amount of these. SQDG are known to be degraded by bacteria in a first step to sulfoquinovose (SQ) (Figure 1A) [6]. SQ is one of the most abundant organically bound sulfur compounds found in nature [7]. SQ can be further degraded, which can lead to a possible final product of H_2_S. SQDG have been described as having anti-cancer activity, though only with the sulfoquinovose (SQ) moiety still bound [8].

Taurine is a further organosulfonate, which is prevalent in the gut. Taurine is derived from exogenous as well as endogenous sources. Exogenous dietary taurine is mainly found in animal products, especially meat and fish [9]. An endogenous source of taurine in the intestinal tract is host-derived conjugated bile acids [10]. A number of bacteria in the gut are able to cleave taurine from these conjugated bile acids, thereby releasing it into the gut [11]. Taurine can be metabolized with H_2_S as a possible final product. A myriad of important roles for the host have been linked to taurine. Taurine is known to be involved in osmoregulation [12], protection from cardiac dysfunction [13] and renal development, and has anti-oxidant properties. In addition, taurine has anti-inflammatory effects, which improve diabetes [14], and may have a crucial role in regulating insulin release [15].

H_2_S, the final product in sulfonate degradation, is a highly reactive gas with a number of physiological effects [17]. H_2_S acts as a neurotransmitter, may be involved in mediating inflammatory responses [3], reduces intestinal motility [18], is involved in gastroprotection [19], is linked to vascular tone [20], and has a cardioprotective effect [21]. H_2_S is also a potent genotoxin, has been associated with a number of detrimental effects [22], and may contribute to colorectal cancer [23] as well as inflammatory bowel disease [24].

Therefore, understanding how sulfonates are metabolized by the intestinal microbiota, which bacterial strains are involved, and how a change in the community structure could alter this are imperative. To facilitate this, the establishment of a method to detect and quantify sulfonates in the intestinal microbiota is critical. Therefore, we developed a method to detect and quantity five sulfonates by a multiplexed, targeted, multiple-reaction-monitoring approach using high-pressure liquid chromatography coupled to tandem mass spectrometry (LC-MS/MS-MRM) in in vivo-derived intestinal microbiome samples. The sulfonates implemented in the assay were taurine and SQ, as well as cysteate, a precursor to taurine [25]; isethionate, a degradation product of taurine [26]; and the SQ degradation product 2,3-dihydroxy-1-propanesulfonate (DHPS) [27] (Figure 1).

As an application pilot study, we investigated how taurine and SQ are utilized in the simplified human intestinal microbiota (SIHUMI) consortium [28], an in vitro simplified human intestinal microbiota model, cultivated with and without *Bilophila wadsworthia*, a known sulfonate utilizer. The SIHUMI consortium consists of eight bacterial species found in the gut, which cover the most important functions of the intestinal microbiota and cover the four most prevalent bacterial phyla in the gut, namely, Actinobacteria, Bacteroidetes, Firmicutes and Proteobacteria. The consortium consists of the species *Anaerostipes caccae, Bifidobacterium longum, Blautia product, Bacteroides thetaiotaomicron Clostridium butyricum, Clostridium ramosum, Lactobacillus plantarum* and *Escherichia coli*. In addition, sulfide, the final degradation product, was also quantified.

## 2. Results

### 2.1. Method Optimization and Validation

The optimized parameters for MS/MS are described in the Materials and Methods. Full-scan MS and corresponding fragment scans for the five sulfonates were measured in negative ionization mode. One transition for each sulfonate was chosen as a quantifier based on signal strength and stability (Table 1). The transition mass-to-charge value picked as a quantifier for cysteate was 168.2 to 151 Da, for isethionate was 125.3 to 95 Da, for DHPS was 155 to 95 Da, for SQ was 243 to 123 Da, and for taurine was 124 to 81 Da.

**Figure 2:**
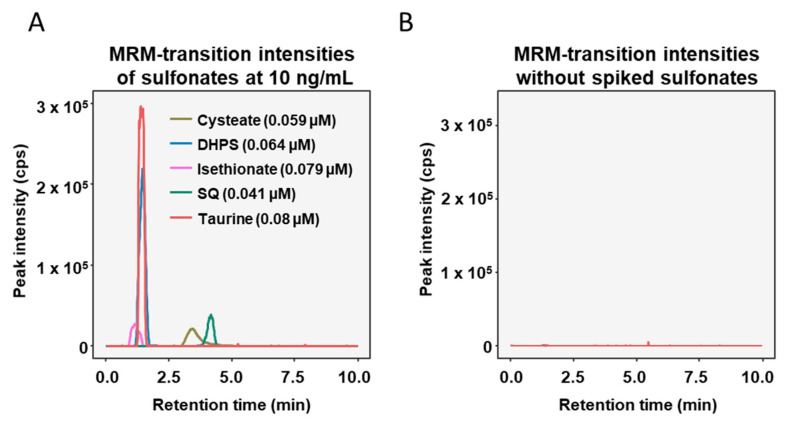
Chromatograms of diluted fecal supernatants spiked with 10 ng/mL of each sulfonate (**A**) and diluted fecal supernatants with no sulfonates added (**B**).

The applicability of the new LC-MS/MS-MRM method was assessed with standards of the sulfonates spiked in human fecal supernatants, which were diluted to 1:10,000 with 50% acetonitrile in water. The optimized LC method was able to separate the sulfonates, except for taurine and DHPS, which had very similar retention times (Figure 2A). In the non-spiked fecal supernatant samples interfering peaks were absent at the chosen transitions (Figure 2B).

The lowest level of detection (LLOD) and lowest level of quantification (LLOQ), defined as the lowest concentrations that can be discriminated from the background with signal-to-noise ratios greater than 3 and 10, respectively, were determined (Table 2). The upper limit of quantification (ULOQ) was given by the maximum concentrations used for the calibration curves (Table 2).

**Figure 3:**
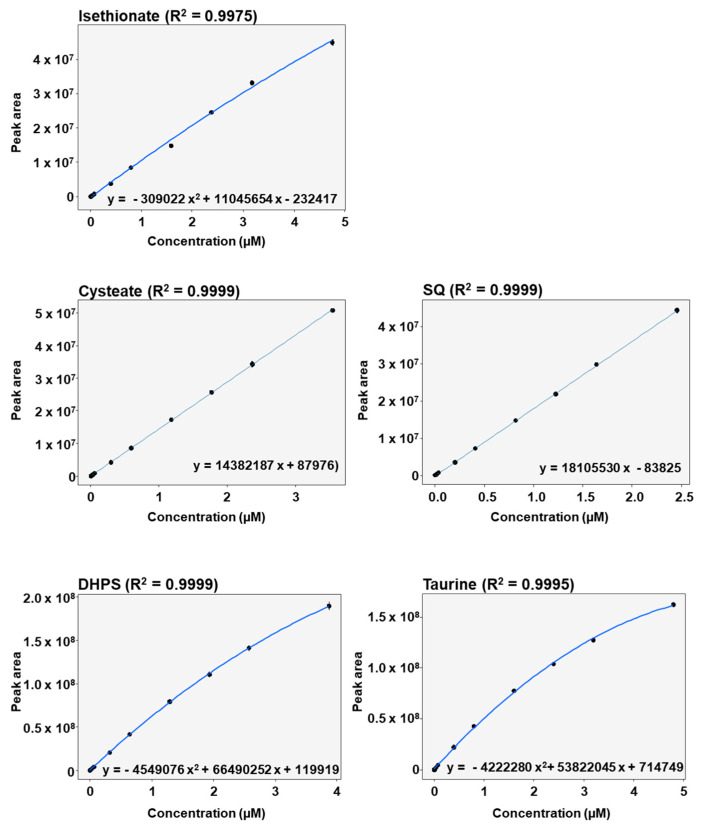
Calibration curves with regression equations and Pearson coefficients of sulfonate standards spiked into fecal supernatants and diluted 1:10,000.

All the calibration curves were measured in seven technical replicates. A linear response was observed for cysteate (R^2^ = 0.9999) and for SQ (R^2^ = 0.9999) in the range of LLOQ to ULOQ, with satisfactory Pearson regression coefficients (Figure 3). Isethionate (R^2^ = 0.9975), DHPS (R^2^ = 0.9999) and taurine (R^2^ = 0.9995) displayed non-linear responses in the range of LLOQ to ULOQ, which was accurately modelled using quadratic polynomial functions (Figure 3). Thus, the method proved to be appropriate for simultaneously measuring the five sulfonates in complex fecal matrices.

The intraday as well as interday accuracy and precision of the measurements were assessed using sulfonates spiked into fecal supernatants at five different concentrations, four of which were according to GLP guidelines, as laid out by the European guidelines (www.ema.europa.eu). These concentrations were the LLOQ, three times the LLOQ, six times the LOQ, 50% of the quantitation range and, finally, 75% of the quantitation range. Seven biological replicates were measured.

The accuracy was calculated as the percentage of the measured concentration compared to the known spiked-in concentrations. These recovery rates as mean relative percentages were determined (Table 3). Acceptable mean recovery values were set at 80% to 120%, as stated by GLP guidelines. For DHPS (94.4%–111.1%), taurine (91.2–116.6%), cysteate (87–106.7%) and SQ (92.3–114.1%), the intra- and interday mean recovery rates were within acceptable values (Table 3). Isethionate also had acceptable mean recovery rates for intraday measurements, except at the LLOQ, where the value was slightly lower than the limit (73.3%). For isethionate, the interday recovery rates were slightly higher than the acceptable levels (128.1–133.2%).

Precision was assessed by the relative standard deviation (RSD). Following GLP guidelines, an RSD value under 20% at the LLOQ and under 15% at higher concentrations was deemed as acceptable. Both the intraday and interday RSD values for DHPS, cysteate and SQ were within the RSD limits or marginally above (Table 3). The intraday RSD was also within acceptable limits for taurine and isethionate. For taurine, the interday RSD value of 3.6 µM (75% of the quantitation range) was a little higher than acceptable (28%). The interday RSD for isethionate was also marginally higher than acceptable for the values at 0.0024 µM (the LLOQ; RSD = 20.3%) and at 3.57 µM (75% of the quantitation range; RSD = 15.5%), whereas for the middle three concentrations, the RSD values were between 18.8% and 22.6%.

### 2.2. Application: The Fate of SQ in the SIHUMI Consortium with and without Bilophila wadsworthia

For a first quantitative analysis of sulfonates, using our method, we set up an experiment using a simplified human intestinal microbiota (SIHUMI) model consortium, which has previously been published [28]. SIHUMI and SIHUMI with *B. wadsworthia* (SIHUMI + Bw) consortia were cultured anaerobically. The bacterial cultures were incubated with either 4 mM SQ or 20 mM taurine. The sulfonate concentrations were measured at 0, 3, 24 and 48 h of incubation. SIHUMI cultures with or without *B. wadsworthia*, which were not incubated with sulfonates, were used as negative controls. As substrate controls, culture medium without bacteria containing either 4 mM SQ or 20 mM taurine was incubated and analyzed at 0 and 48 h.

**Figure 4:**
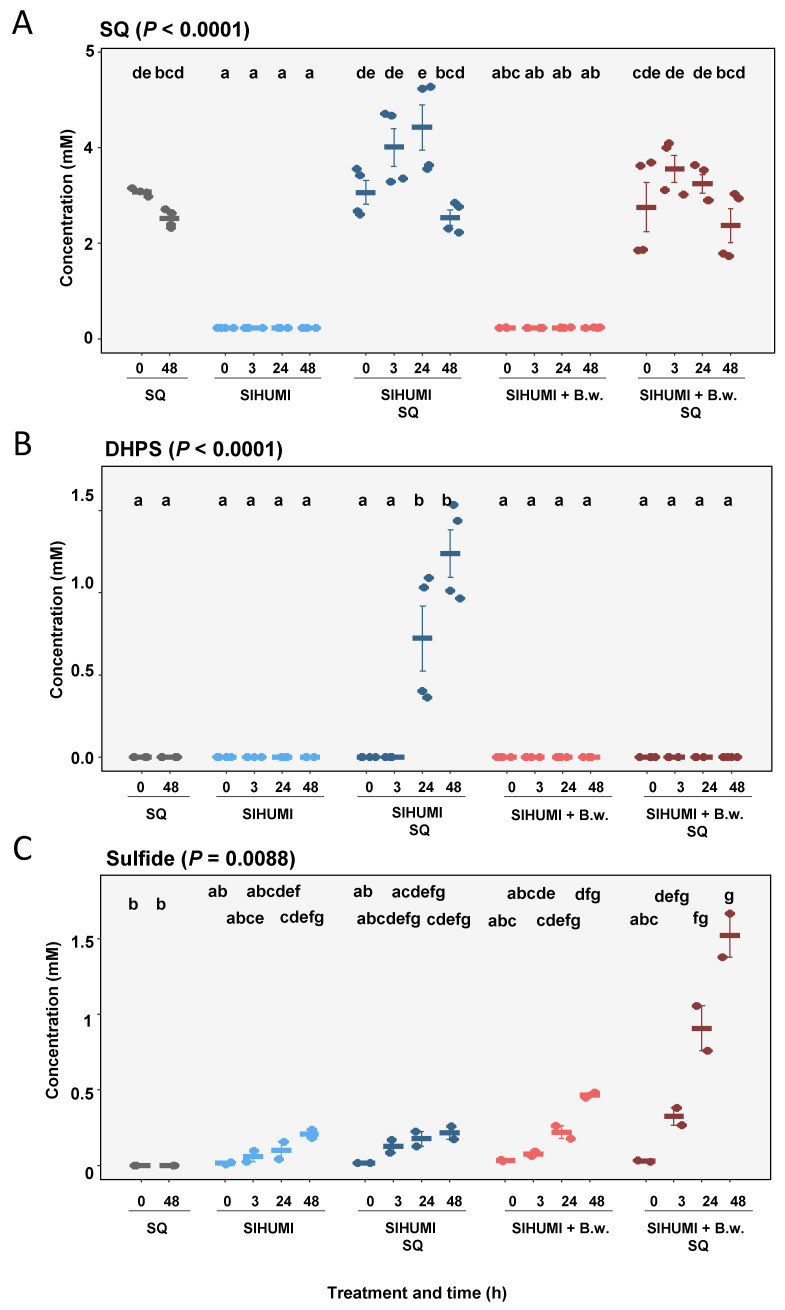
Mean concentrations of SQ (**A**), DHPS (**B**) and sulfide (**C**) at 0, 3, 24 and 48 h in substrate control with SQ, simplified human intestinal microbiota (SIHUMI), SIHUMI with SQ, SIHUMI and *Bilophila wadsworthia*, and SIHUMI and *Bilophila wadsworthia* with SQ. Error is SEM. *P* calculated according to Kruskal–Wallis tests with post hoc Dunn tests. Differing letters depict significant differences between groups.

In the substrate controls, we observed no significant changes in the concentration of SQ (0 h: 3.7 ± 0.04 mM; 48 h: 2.52 ± 0.09 mM) (Figure 4A) and detected no DHPS at any of the time points (Figure 4B). The SIHUMI culture incubated with SQ exhibited a slight increase in SQ from 0 h (3.07 ± 0.25 mM) to 24 h (4.01 ± 0.40 mM) after spike in, though this was not significant (Figure 4A). This was followed by a significant decrease in SQ from 24 h (4.43 ± 0.48 mM) to 48 h (2.54 ± 0.31 mM). DHPS was first observed after 24 h (0.72 ± 0.20 mM) in the SQ-incubated SIHUMI culture, with an increase in concentration detected after 48 h (1.24 ± 0.14 mM) (Figure 4B). The quantification of sulfide in the bacterial incubations of the SIHUMI consortium with or without *B. wadsworthia* and SQ (4 mM) revealed that sulfide was solely quantitatively formed when *B. wadsworthia* was present. However, SQ was only partly converted to sulfide, which was detected at 1.8 mM (Figure 4C).

### 2.3. Application: The SIHUMI Consortium Is Only Able to Degrade Taurine to Sulfide in the Presence of Bilophila wadsworthia

**Figure 5:**
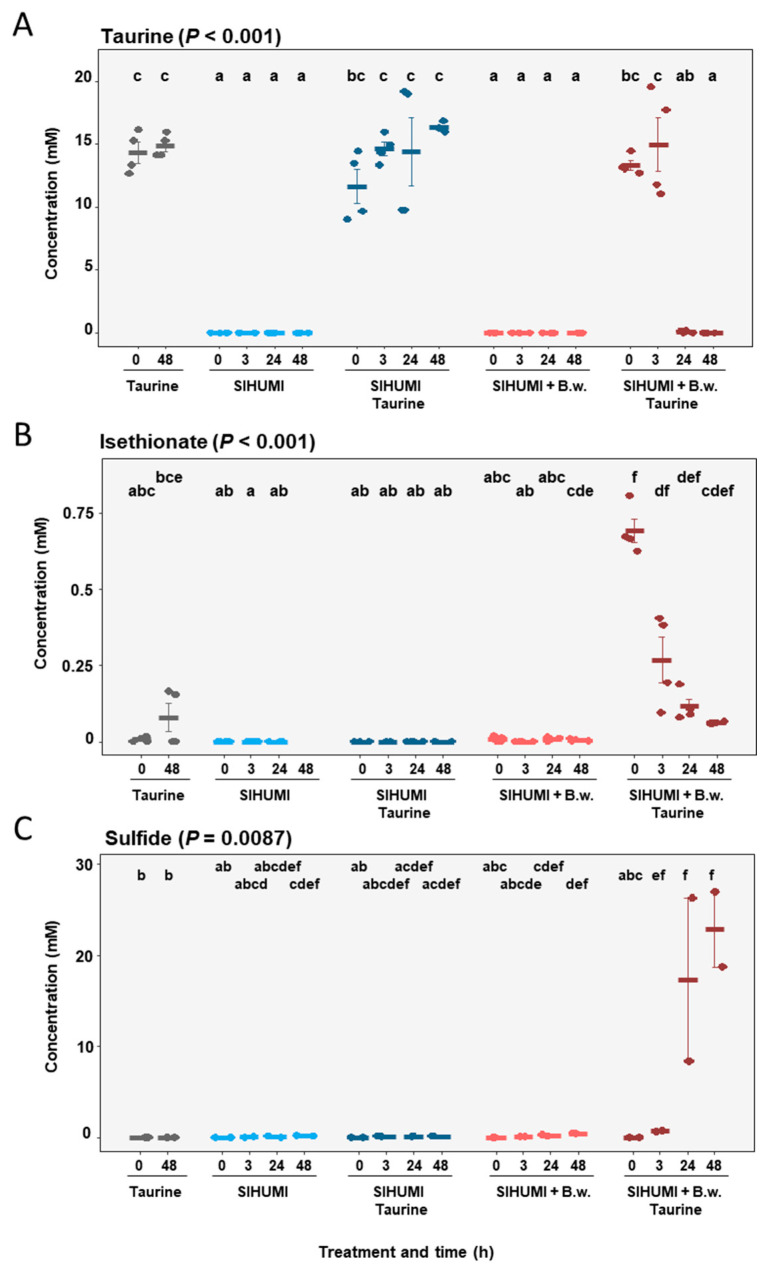
Mean concentrations of taurine (**A**), isethionate (**B**) and sulfide (**C**) at 0, 3, 24 and 48 h in substrate control with taurine, SIHUMI, SIHUMI with taurine, SIHUMI and *Bilophila wadsworthia*, and SIHUMI and *Bilophila wadsworthia* with taurine. Error is SEM. *P* calculated according to Kruskal–Wallis tests with post hoc Dunn tests. Differing letters depict significant differences between groups.

The SIHUMI cultures incubated with taurine displayed no significant changes in taurine concentration at any time point measured (0 h: 11.62 ± 1.35 mM; 3 h: 14.60 ± 0.55 mM; 24 h: 14.39 ± 2.69 mM; 48 h: 16.31 ± 0.18 mM) (Figure 5A). Furthermore, no isethionate or sulfide was detected for these samples (Figure 5B).

By contrast, SIHUMI + Bw cultures incubated with taurine exhibited a substantial decrease in taurine concentration within 24 h, with no detection of taurine after 48 h (0 h: 13.30 ± 0.39 mM; 3 h: 14.98 ± 2.12 mM; 24 h: 0.076 ± 0.04 mM; 48 h: not detected) (Figure 5A). Isethionate was detected in SIHUMI + Bw incubated with taurine, with a decrease in concentration at 0, 3, 24 and 48 h observed (Figure 5B). In addition, sulfide was detected after 3 h and increased to a concentration of 22.8 mM after 48 h, revealing a complete reduction of taurine to sulfide (Figure 5C).

## 3. Discussion

Previously, several methods have been used to analyze sulfonates in complex biological fluids. Denger et al. analyzed sulfonates in incubations with bacterial cultures also using LC-MS/MS [6]. Here, sulfonates, including SQ and DHPS, were separated by hydrophilic interaction liquid chromatography and then detected by mass spectrometry, though an absolute quantification was not performed [6]. SQ has also been detected by NMR, though not absolutely quantified [8]. Earlier studies have detected taurine after derivatization by chromatography coupled to UV-absorption detection [29]. In other studies, cysteate and isethionate were also analyzed by chromatography coupled to UV-absorption detection following derivatization [30,31]. The absolute quantification of taurine is part of a commercially available kit, which analyzes 180 metabolites using an LC-MS/MS-MRM method [32]. The newly developed LC-MS/MS MRM method presented herein exhibited a reproducible, accurate and precise absolute quantification of five sulfonates relevant in understanding the fate of sulfur-containing compounds in the gut. Furthermore, sample preparation is simple and inexpensive and does not require the derivatization of compounds.

We applied the developed method to investigate SQ and taurine degradation by the SIHUMI model consortium either with or without *B. wadsworthia*. To date, two pathways of bacterial SQ degradation have been described. The first, which was discovered in *Pseudomonas putida* SQ1, is an Entner–Doudoroff-type pathway, by which SQ is degraded via a number of intermediates to sulfolactate, which is then exported [16] (Figure 1A). The second is the sulfoglycolysis pathway, which was identified in *Escherichia coli* K-12 [6]. In this pathway, SQ is degraded to the final product DHPS (Figure 1A), which is then exported out of the cell. The SIHUMI consortium used in our study harbored *E. coli* K12. The formation of DHPS in the SIHUMI cultures incubated with SQ is consistent with sulfoglycolysis. SQ degradation is not known for any of the other members of the SIHUMI consortium according to the Biocyc data repository [33] or the Kyoto Encyclopedia of Genes and Genomes (KEGG) database [34]. Interestingly, we observed no DHPS in the SIHUMI plus *B. wadsworthia* consortium incubated with SQ, although we did observe a strong decrease in SQ. This suggests that the DHPS formed and excreted by *E. coli* was further utilized by *B. wadsworthia*, which is known to be able to degrade a number of sulfur-containing compounds to dihydrogen sulfide [17]. However, *B. wadsworthia* is not listed as being able to degrade SQ based on Biocyc and KEGG database entries. In a recent study, the pathway of DHPS degradation to dihydrogen sulfide by *Desulfovibrio sp.* strain DF1 was deciphered [27]. Notably, the authors of this study also identified the genes encoding key enzymes involved in this pathway in the three publicly available genomes of *B. wadsworthia* strains [27]. This fits well with the higher concentrations of sulfide detected at the later time points in SIHUMI with *B. wadsworthia* than in SIHUMI. In addition, the sulfide levels detected at 24 and 48 h in the SIHUMI with *B. wadswortia* were approximately equal to the amounts of the DHPS detected in SIHUMI without *B. wadsworthia*. Taken together, this suggests that in the SIHUMI plus *B. wadsworthia* consortium incubated with SQ, *B. wadsworthia* completely degrades the DHPS formed by *E. coli* to sulfide.

In our second study, where we incubated SIHUMI as well as SIHUMI with *B. wadsworthia* with taurine, the SIHUMI culture revealed no change in taurine concentration. Hence, no utilization of taurine by the SIHUMI community members took place. A number of bacteria are known to utilize taurine, including *E. coli* and *B wadsworthia* [22,35]. However, *E. coli* metabolizes taurine when sulfate is lacking and only under aerobic conditions [36]. The SIHUMI culture was incubated with taurine under anaerobic conditions, and therefore, it is likely that the *E. coli* did not utilize taurine. The entries for the other members of the SIHUMI culture in the Biocyc and in the KEGG depository reveal that none had a known metabolic pathway for degrading taurine. In the SIHUMI consortium with *B. wadsworthia*, the taurine concentration decreased and isethionate was detected. Isethionate is an intermediate in the sulfoacetaldehyde degradation IV pathway according to Metacyc (https://metacyc.org/), which is part of the Biocyc depository. Previously, *B. wadsworthia* was shown to use this pathway for taurine degradation [22] (Figure 1B). In *E. coli*, during sulfate-starvation conditions, taurine is degraded by a different pathway, which leads to aminoacetalaldehyde and sulfide formation [36]. Taken together, in our experimental setup, *B. wadsworthia* solely utilized taurine in the nine-membered consortium, since only in the presence of *B. wadsworthia* was taurine degraded and were isethionate and sulfide detected.

## 4. Materials and Methods

### 4.1. Chemicals and Reagents

Acetonitrile was purchased from Sigma-Aldrich (St. Louis, MO, USA). All the solvents for mass spectrometry were of analytical grade purity. Water (resistivity, 18.2 MΩcm) was purified using a Milli-Q System (Millipore, Milford, MA, USA). Formic acid was purchased from Honeywell Fluka (Muskrgon, MI, USA). Cysteic acid and isethionic acid were purchased from Sigma-Aldrich (St. Louis, MO, USA). 2,3-dihydroxypropane-1-sulfonate and sulfoquinovose were purchased from MCAT GmbH (Donaueschingen, Germany). Taurine was purchased from Roth GmbH (Karlruhe, Germany). The solutions, and their chemical compositions, used for cultivation are listed in Table 4.

### 4.2. Collection and Processing of Fecal Samples

Fecal samples of a human donor were collected as part of a study approved by the ethics committee of the University of Potsdam, Germany (no. 11/2016). The first fecal sample of the day was collected using a feces catcher (Servoprax, Germany). Three aliquots of different fecal areas were immediately transferred to a fecal collection tube (Sarstedt, Germany) with a perforated lid. The tube was kept under anoxic conditions in a plastic box containing an AnaeroGen sachet (Thermo Fisher Scientific, Germany) at 4 °C. Further processing was performed in an anaerobic chamber (MACS Anaerobic Workstation, Don Withley Scientific, UK). One gram of the fecal sample was homogenized in anoxic phosphate-buffered saline (PBS, pH 7.0, Table 4) by vortexing with glass beads (c. 3 mm; Roth, Germany) to yield a 10% fecal suspension. The fecal suspension was further diluted to 1% in a Hungate tube containing anoxic PBS supplemented with 3.18 mM sterile filtered Ti(III) nitrilotriacetate (Sigma-Aldrich, Germany) as a reductant. Subsequently, the fecal slurry aliquots were centrifuged (14,000× *g*, 4 °C, 5 min) and the supernatant frozen until further processing.

For spiking experiments, the fecal supernatants were serially diluted using 50% acetonitrile and 0.1% formic acid in water to obtain final dilutions of 1:10,000.

### 4.3. Replicates

For the validation of the LC-MS/MS-MRM method and construction of calibration curves, we used seven technical replicates. To determine the robustness, accuracy and precision of the method, five different concentrations of sulfonates were spiked into fecal supernatants. For each concentration, seven biological supernatant replicates were prepared.

### 4.4. LC-MS/MS-MRM Method

The five sulfonates, cysteic acid, isethionic acid, 2,3-dihydroxypropane-1-sulfonate, sulfoquinovose and taurine, were analyzed using multiple reaction monitoring with LC-MS/MS. Sample aliquots (15 µL) were injected into the Ultimate 3000 HPLC (Dionex, Sunnyvale, CA, USA) and separated on a BEH amide column (2.1 × 100 mm, 1.7 µm; Waters, Milford, NZ, USA). The column temperature was kept at 60 °C, and the flow rate of the elution solvents was 0.5 mL/min. The elution solvents used were A: 50% acetonitrile and 0.1% formic acid in water, and B: 0.1% formic acid in water. The LC gradient was run for 2 min at 80% A followed by a linear gradient of 3 min to 50% A, which was held for 2 min. This was followed by a rapid increase in A to 80%, which was held for a further 3 min to equilibrate the column.

The sulfonates were identified and quantified using specific MRM traces for the five analytes measured on a QTRAP^®^ 5500 (AB Sciex, Framingham, MA, USA) in negative mode. The ionization source settings were as follows: ion spray voltage of −4.5 kV, temperature of 500 °C, curtain gas flow of 30, collision gas/medium and ion source gases of 40 and 60. The transition masses, corresponding declustering potentials and collision energies were determined by direct infusion prior to flow injection analysis (Table 1). Data acquisition and analysis were performed using the Analyst^®^ Software (Version 1.6.2, AB Sciex).

### 4.5. Method Validation

Standard curves were prepared using fecal supernatants as matrices spiked with five sulfonates of identical concentration. The method validation included an analysis of the linearity, limit of detection (LOD), limit of quantification (LOQ), accuracy and precision (intra- and inter-assay variation). The standard curves were generated by linear regression (y = ax + b) as matrix-assigned calibrations or, if linearity was not met, as a polynomial function (y = ax2 + bx+ c). The calibration curves were generated using eleven concentrations: 0.1, 0.5 1, 5, 10, 50, 100, 200, 300, 400 and 600 ng/mL (n = 7) in 1:10,000 fecal supernatant dilutions. The LOD and LOQ were estimated by the lowest concentrations of spiked sample with signal-to-noise ratios of at least 3 and 10, respectively. The spiked samples were prepared with the following concentrations after 1:10,000 dilution: 0.25, 0.5, 0.75, 1, 1.5, 2, 3, 6, 10, 20, 50,100, 200, 300, 450 and 600 ng/mL. The intraday and interday accuracy, defined as the recovery in percent, and precision, defined as relative standard deviation (RSD) in percent, were calculated at five different concentrations in the fecal supernatants.

### 4.6. Bacterial Growth Conditions

*Bilophila wadsworthia* DSM 11,045 was purchased from the German Collection of Microorganisms and Cell Cultures (DSMZ, Braunschweig, Germany). *B. wadsworthia* was grown at 37 °C under anoxic conditions in a liquid culture medium adapted from da Silva et al. (DS medium) [37], which contained 19 mM NH_4_Cl, 17 mM NaCl, 2 mM MgCl_2_ x 6 H_2_O, 7 mM KCl, 0.3 mM CaCl_2_ x 2 H_2_O, 1 mM K_2_HPO_4_, 40 mM sodium DL-lactate, 40 mM sodium formate, 3.5 mg/L yeast extract, 1 mL/l selenite–tungstate solution (see Table 4), 1 mL/L trace element solution (see Table 5), 1.2 µM 1,4-naphthoquinone and 2 µM resazurin. The medium was adjusted to pH 7.4, gas flashed with N_2_/CO_2_ (80/20, *v*/*v*) and autoclaved in gas-tight Hungate tubes. Directly before use, sterile filtered 0.5 mM Ti(III) nitrilotriacetate solution (see Table 4) [38], 1 mL/L 7-vitamin solution (see Table 4) and 30 mM NaHCO_3_ were added to the medium. The DS medium used for the cultivation of *B. wadsworthia* was additionally supplemented with 20 mM sterile filtered gas-flashed (N_2_/CO_2_, 80/20, *v*/*v*) taurine, whereas the medium for the incubation experiments was free of taurine (if not indicated otherwise).

The SIHUMI consortium consists of Anaerostipes caccae DSM 14662T, Bacteroides thetaiotaomicron DSM 2079T, Bifidobacterium longum NCC 2705, Blautia product DSM 2950T, Clostridium butyricum DSM 10702T, Clostridium ramosum DSM 1402T, Escherichia coli K-12 MG1655 and Lactobacillus plantarum DSM 20174T. The bacteria from the strain collection of the German Institute of Human Nutrition Potsdam-Rehbruecke (DIfE) were individually cultivated anoxically at 37 °C in Brain Heart Infusion (BHI, Roth) broth, supplemented with 5 g/L of yeast extract and 5 mg/L of hemin (Serva) (YH-BHI medium). To grow Clostridium ramosum, YH-BHI medium was mixed in equal parts with Yeast Casitone Fatty Acid (YCFA) medium (DSMZ medium 1611).

### 4.7. Bacterial Incubation Experiments

SIHUMI bacteria and *B. wadsworthia* were individually cultivated as described above, and the cell numbers of overnight cultures were determined using a Thoma counting chamber. The ratio of the cell numbers of individual bacteria were adopted from cell counts present in the cecal contents of SIHUMI mice additionally colonized with *B. wadsworthia* (Table 5; unpublished data). For each 10 mL incubation, DS medium supplemented with either taurine (20 mM) or sulfoquinovose (SQ, 4 mM) was inoculated with the SIHUMI bacteria or the SIHUMI consortium and *B. wadsworthia*. Control incubations of medium containing only sulfonates or bacteria were included. The incubations were conducted under anoxic conditions at 37 °C in duplicate. All incubations and controls were performed in duplicate (*n* = 2). Samples (600 µL) were withdrawn after 0, 3, 24 and 48 h for the quantification of sulfide, SQ, 2,3-dihydroxypropane-1-sulfonate (DHPS), taurine and isethionate. The sulfide assay was performed immediately after sampling. For the analysis of sulfonates by LC-MS/MS-MRM, an aliquot of 250 µL for each sample was centrifuged (14,000× *g*, 4 °C, 5 min), and 50 µL of the supernatant weas stored at −20 °C until further processing.

### 4.8. Bacterial Sample Preparation for Sulfonate Quantification

Samples were thawed and centrifuged at 18,000× *g* at room temperature (RT) for 2 min, and the supernatants were diluted 1:10,000 in 50% aqueous acetonitrile. Subsequently, 500 µL of each sample was placed in a glass vial (Wicom, Heppenheim, Germany) and stored at −80 °C until LC-MS/MS analysis was performed with two technical replicates.

### 4.9. Sulfide Quantification

Sulfide concentrations were determined photometrically by the methylene blue method [39]. Sample volumes of 250 µL (if required, diluted with water) were added to 1.5 mL plastic tubes containing 25 µL of 100 mM NaOH and 5 µL of 327 mM zinc acetate solution and vortexed. Then, 20 µL of detection reagent (16 mM N,N-dimethyl-1,4-phenylenediamine and 22 mM Fe (III) chloride dissolved in 100 mL of 18.5% aqueous HCl) was added and incubated for 20 min at room temperature (RT). Subsequently, the mixtures were centrifuged (12,000× *g*, RT, 3 min), and 200 µL aliquots of the supernatants were transferred to 96-well plates for photometric measurement at 670 nm (Infinite M200 PRO, Tecan, Männedorf, Switzerland). Concentrations were calculated based on a calibration curve obtained with Na_2_S dissolved in water/100 mM NaOH/327 mM zinc acetate solution (50:5:1, *v*/*v*/*v*) in the range of 0–200 mM.

### 4.10. Statistics for Application Experiment

The statistics were calculated and figures produced using an in-house-written R script [40] and using the R package ggplots2 [41]. For statistics, the Kruskal–Wallis test was used for determining significant differences between groups, with post hoc pairwise significance determined by Dunn tests. Results were regarded as statistically significant for *P* < 0.05. All errors reported are the standard errors of the mean.

### 4.11. LC-MS/MS-MRM Data Availability

The raw LC-MS/MS-MRM data were deposited in the Metabolomics Workbench (www.metabolomicsworkbench.org) data repository under the project ID PR001014 (study IDs: ST001498, ST001497 and ST001496).

## 5. Conclusions

We were able to establish a method to quantify SQ, DHPS, cysteate, taurine and isethionate in intestinal microbiota samples. We were able to show its practicality in a pilot study by incubating a simplified intestinal microbiota model system with either SQ or taurine and analyzing the sulfonates. With this established method, it will now be possible to investigate and follow the fate of these sulfonates in the human gut microbiome or in intestinal microbiota model systems, and thereby make predictions on sulfide production in the gut, which has serious health implications.

## Figures and Tables

**Table 1:** Multi reaction monitoring (MRM)ransitions and settings for sulfonate measurements (* transitions used as quantifiers). DP, declustering potential; CE, collision energy. Ionization mode was negative.

Sulfonate	Q1 Mass (Da)	Q3 Mass (Da)	Time (ms)	ID	DP (V)	CE (V)
Cysteate	168.2	151	20	Cysteate_1 *	−80	−17
	168.2	86	20	Cysteate_2	−80	−18
	168.2	81	20	Cysteate_3	−80	−27
	168.2	71	20	Cysteate_4	−80	−24
Isethionate	125.3	107	20	Ise_1	−90	−21
	125.3	95	20	Ise_2 *	−90	−20
	125.3	80	20	Ise_3	−90	−32
2,3-dihydroxy-1-propanesulfonate	155	95	20	DHPS_1 *	−96	−24
	155	80	20	DHPS_2	−96	−39
Sulfoquinovose	243	123	20	SQ_1 *	−110	−31
	243	95	20	SQ_2	−110	−44
	243	153	20	SQ_3	−110	−24
	243	183	20	SQ_4	−110	−23
	243	81	20	SQ_5	−110	−32
Taurine	124	81	20	Taurine_1 *	−95	−30
	124	80	20	Taurine_2	−100	−20
	124	65	20	Taurine_3	−100	−19

**Table 2:** Level of detection (LOD), lower level of quantitation (LLOQ) and upper level of quantitation (ULOQ) of sulfonates in fecal supernatants analyzed at 1:10,000 dilution (1st, 2nd and 3rd columns); molar mass of sulfonates; and LLOQ and ULOQ of sulfonates in fecal supernatants before extraction and dilution.

	In Extracted Diluted Measured Sample				In Fecal Supernatant	
Sulfonate	LLOD (µM)	LLOQ (µM)	ULOQ (µM)	Molar Mass (g/mol)	LLOQ (mM)	ULOQ (mM)
Cysteate	0.0015	0.003	3.55	169.15	0.03	35.5
Isethionate	0.0079	0.024	4.76	126.13	0.24	47.6
2,3-dihydroxy-1-propanesulfonate	0.0006	0.0016	3.87	155.15	0.016	38.7
Sulfoquinovose	0.001	0.002	2.46	244.22	0.02	24.6
Taurine	0.001	0.002	4.8	125.14	0.02	48.0

**Table 3:** Mean recovery and intraday and interday relative standard deviation of sulfonates spiked into fecal supernatants and diluted 1:10,000. (RSD relative standard deviation)

Sulfonate	Theoretical Concentration (µM)	Intraday Mean Recovery (%)	Intraday RSD (%)	Interday Mean Recovery (%)	Interday RSD (%)	N
2,3-dihydroxy-1-propanesulfonate	0.0016	95.2	8.2	106.2	8.1	7
	0.0048	94..4	9.4	104.4	8.7	7
	0.0097	94.4	9.3	106.9	5.1	7
	1.933	96.7	6.5	107.2	11.2	7
	2.9	96	12.1	111.1	12.9	7
Taurine	0.002	102.9	5.5	111.6	5.5	7
	0.006	104.4	6.5	113.6	10.7	7
	0.012	103.4	6,3	116.6	10.4	7
	2.4	95.9	2.4	106.7	9.3	7
	3.6	91.2	9.9	115	28	7
Cysteate	0.003	87	15.7	95.8	9.9	7
	0.009	89.1	11.1	98..4	4.6	7
	0.0177	95.5	6.9	98.5	11.4	7
	1.773	97.1	4.8	106.7	8.3	7
	2.66	94.8	8.2	103.9	5	7
Isethionate	0.024	73.3	9	129.3	20.3	7
	0.079	79.1	8.8	133.2	22.1	7
	0.396	81.4	4.8	128.1	22.6	7
	2.37	85.5	7.2	117.8	18.8	7
	3.57	79.7	9.1	114.3	15.5	7
Sulfoquinovose	0.002	94.8	20	109.4	15.5	7
	0.006	94.8	7.1	103.3	11.9	7
	0.012	92.3	7.6	104.1	15.5	7
	1.228	98.1	3.4	114.1	15.9	7
	1.843	96.6	6.4	112	14.3	7

**Table 4:** Composition of solutions used. If not indicated otherwise, all solutions were prepared with pure distilled water (Ultra Clear, Siemens Water Technologies, Günsburg, Germany) and chemicals and solvents were purchased from Fluka (Muskrgon, MI, USA).

Solution	Components with Concentration
Anoxic phosphate-buffered saline (PBS)	8.5 g/L NaCl
	0.3 g/L KH_2_PO_4_
	0.6 g/L Na_2_HPO_4_
	0.1 g/L bacteriological peptone
	1 mg/L resazurin
	40 mM sodium DL-lactate
	40 mM sodium formate
	pH 7.0
	N_2_/CO_2_ (80/20, *v/v*) as gas phase,
	autoclaved at 121 °C for 15 min
Trace-element solution	10 mL/L HCl
	1.5 g/L FeCl_2_ × 4 H_2_O
	70 mg/L ZnCl_2_
	100 mg/L MnCl_2_ × 4 H_2_O
	6 mg/L H_3_BO_3_
	190 mg/L CoCl_2_ × 6 H_2_O
	2 mg/L CuCl_2_ × 2 H_2_O
	24 mg/L NiCl_2_ × 6 H_2_O
	36 mg/L Na_2_MoO_4_ × 2 H_2_O
Selenite–tungstate solution	500 mg/L NaOH
	3 mg/L Na_2_SeO_3_ × 5 H_2_O
	4 mg/L Na_2_WO_4_ × 2 H_2_O
Seven-vitamin solution	100 mg/L Vitamin B_12_
	80 mg/L p-amino benzoic acid
	20 mg/L D (+)-biotin
	200 mg/L nicotinic acid
	100 mg/L calcium pantothenate
	300 mg/L pyridoxine hydrochloride
	200 mg/L thiamine hydrochloride × 2 H_2_O
Ti(III) nitrilotriacetate solution	19.2 g/L nitrilotriacetic acid diluted in anoxic distilled water,
	pH of 9 adjusted with NaOH
	19.2 mL 20% TiCl_3_ (Acros),
	pH of 7 adjusted with Na_2_CO_3_ (80 g/L)

**Table 5:** Bacterial strains used and cell numbers transferred to medium.

Bacterial Species	Strain Designation (^T^ Type Strain)	Cell Number in 10 mL DS Medium
*Anaerostipes caccae*	DSM 14662^T^	10^7^
*Bifidobacterium longum*	NCC 2705	10^6^
*Blautia producta*	DSM 2950^T^	10^9^
*Bacteroides thetaiotaomicron*	DSM 2079^T^	10^10^
*Clostridium butyricum*	DSM 10702^T^	10^7^
*Clostridium ramosum*	DSM 1402^T^	10^9^
*Escherichia coli* K-12	MG1655	10^9^
*Lactobacillus plantarum*	DSM 20174^T^	10^3^
*Bilophila wadsworthia*	DSM 11045	10^9^
